# *Ephedra*-Treated Donor-Derived Gut Microbiota Transplantation Ameliorates High Fat Diet-Induced Obesity in Rats

**DOI:** 10.3390/ijerph14060555

**Published:** 2017-05-23

**Authors:** Jing-Hua Wang, Bong-Soo Kim, Kyungsun Han, Hojun Kim

**Affiliations:** 1 Department of Rehabilitation Medicine of Korean Medicine, Dongguk University, 814 Siksa, Goyang, Gyeonggi-do 10326, Korea; ewccwang@gmail.com (J.-H.W.); latortue@hanmail.net (K.H.); 2 Key Laboratory of Xin’an Medicine, Ministry of Education, Anhui University of Traditional Chinese Medicine, Meishan Road 103, Hefei 230038, China; 3 Department of Life Sciences, Hallym University, Chuncheon, Gangwon-do 24252, Korea; bongsoo.km@gmail.com

**Keywords:** ephedra, microbiota, transplantation, high fat diet, obesity

## Abstract

Changes in gut microbiota (GM) are closely associated with metabolic syndrome, obesity, type 2 diabetes and so on. Several medicinal herbs, including *Ephedra sinica* (Es), have anti-obesity effects that ameliorate metabolic disorders. Therefore, in this study we evaluated whether Es maintains its anti-obesity effect through Es-altered gut microbiota (EsM) transplantation. GM was isolated from cecal contents of Es treated and untreated rats following repeated transplants into obese rats via oral gavage over three weeks. High-fat-diet (HFD)-induced obese rats transplanted with EsM lost significant body weight, epididymal fat, and perirenal fat weight, but no remarkable changes were observed in abdominal fat, liver, cecum weight and food efficiency ratio. In addition, treatment with EsM also significantly lowered the fasting blood glucose, serum insulin level, and insulin resistance index. Meanwhile, EsM transplantation significantly reduced gene expression of proinflammatory cytokines interleukin-1 and monocyte chemotactic protein-1. Rats treated with EsM also showed changed GM composition, especially blautia, roseburia and clostridium, significantly reduced the level of endotoxin and markedly increased the acetic acid in feces. Overall, our results demonstrated that EsM ameliorates HFD-induced obesity and related metabolic disorders, like hyperglycemia and insulin resistance, and is strongly associated with modulating the distribution of GM, enterogenous endotoxin and enteral acetic acid.

## 1. Introduction

Obesity was officially recognized as a disease by the American Medical Association in 2013 [1]. According to epidemiological findings from the World Health Organization, obesity, as a typical metabolic disorder, is becoming an intractable issue worldwide [2]. In general, obesity is caused by imbalance of energy intake and expenditure [3]. Therefore, reduction of energy intake and/or increase of basal metabolic rate (BMR) has been simplistically regarded as a therapeutic solution for controlling body weight gain [4]. However, both compulsive calorie restriction and permanent elevation of the BMR are extremely difficult in practice. Even invasive bariatric surgery and pharmacotherapy produce a variety of side effects and complications in Western medicine. Consequently, the need for an effective therapy for weight-loss with fewer or no adverse side effects is urgent.

Further, adipose tissue not only stores fat as a form of lipid but exerts sophisticated endocrine, autocrine, and paracrine functions to maintain energy homeostasis [5]. Large amounts of adipokines secreted by excessive adipocytes, such as interleukin (IL)-10, monocyte chemotactic protein-1 (MCP-1) [6,7], C-reactive protein [8], continuously promote low-grade inflammation resulting in a cascade of metabolic disorders [9]. Thus, obesity is characterized as a chronic and systemic inflammatory disease [10]. In addition, increasing scientific evidence demonstrates that a high-fat-diet (HFD) causes structural alteration of gut microbiota resulting in increased gastrointestinal permeability and endotoxemia [11]. Furthermore, metabolic endotoxemia evidently initiates obesity and insulin resistance [12,13]. That is, gut microbiota play a crucial role in metabolism as well as adipogenesis [14]. Interestingly, after fecal microbiota transplantation from a healthy but overweight donor, body weight gain was observed in a human subject [15]. Recent findings further revealed that gut microbiota impact host metabolism by differential DNA methylation as an epigenetic regulator [16]. Hence, effective modulation of gut microbiota might be a promising strategy for management of metabolic disorders, including obesity.

As a kind of therapeutic intervention, many medicinal herbs, formulas, and herbal derived components, including *Flos lonicera* [17], *Rhizoma coptidis* [18], Gegen Qinlian Decoction (composed of *Radix puerariae*, *Radix scutellariae*, *Rhizoma coptidis*, and honey-fried licorice root) [19], and berberine [20], were verified to exert beneficial efficacy on alleviating obesity and other chronic metabolic disorders via modulation of gut microbiota. *Ephedra sinica* (Es), also named ma huang, is commonly used in Asia as a traditional medicine for the treatment of common cold, flu, asthma, arthralgia, and edema for thousands of years [21]. Currently, Es and its principal active constituent, ephedrine, are commonly used as food additives for reducing body weight worldwide due to its powerful sympathomimetic action [22]. Several studies reported on the effects of Es on body weight loss in both obese human subjects and HFD-induced obese animal models associated with alteration of gut microbiota and regulation of adipokines [23,24]. Nevertheless, it is still undetermined whether Es itself or Es altered gut microbiota (EsM) effectively induce loss of body weight.

Therefore, in the current study we tried to verify the anti-obesity effect of Es through regulation of gut microbiota and to determine how gut microbiota can be modified by Es. We estimated the anti-obesity effect of intestinal microbiota regulated by Es and its possible mechanism.

## 2. Materials and Methods

### 2.1. Aqueous Extraction of Ephedra sinica

Korean pharmacopoeia standard Es was obtained from Dongguk University Ilsan International Hospital (Goyang, Korea). After washing with distilled water (DW) and oven-dying for 12 h, 500 g of Es was boiled in 4 L of DW for 4 h and filtered using a 300-mesh filter (50 μm). The water extract was concentrated using a vacuum rotary evaporator following lyophilization at −70 °C and stored at −20 °C for future use.

### 2.2. High-Performance Liquid Chromatography (HPLC)

HPLC-based fingerprinting was accomplished using two-dimensional HPLC (Agilent HP1200, Agilent Technologies, San Jose, CA, USA). Ephedrine (Sigma-Aldrich, St. Louis, MO, USA), as a principal component of Es, was used as a standard. Briefly, 100 μg of water extract of Es, 10 μg of ephedrine and 200 mg of rat fecal samples from the end of microbiota preparation experiment were dissolved in 1 mL 50% of methanol separately. After sonication and centrifuging, the supernatant of samples were filtrated by 0.45 μm membrane filter (Sartorius AG, Goettingen, Germany) and prepared for HPLC analysis. The Agilent HPLC system consisted of a quaternary pump, on-line solvent degasser, Agilent sample manager/column heater module, and Agilent UV detector. An Agilent Eclipse XDB-C18 (5 mm, 4.6 mm × 250 mm) column was used and the compounds were eluted with solvents composed of acetonitrile: water: phosphoric acid (40:60:0.1 *v*/*v*/*v*) containing sodium lauryl sulfate at a concentration of 5 g/L as an ion-pairing agent. Peak identity of ephedrine in the samples was confirmed by comparison of spectrum and retention time. HP1200 ChemStation software (Agilent Technologies, Santa Clara, CA, USA) was applied and all chromatograms were obtained using a wavelength of 215 nm.

### 2.3. Animals and Experimental Design

The animal experiments were performed according to the international guidelines (Guide for the Care and Use of Laboratory Animals, Institute of Laboratory Animal Resources, Commission on Life Sciences, National Research Council, USA; National Academy Press: Washington DC, 1996). The animal experiment protocols were reviewed and approved by the Institutional Animal Ethical Committee of Dongguk University (approval number: 2013-0580). All surgical procedures were performed under Zoletil (tiletamine-zolazepam, Virbac, Carros, France) and Rompun (xylazine-hydrochloride, Bayer, Leverkusen, Germany) combination (1:1, *v*/*v*) anesthesia. The detailed experimental schedule is as follows.

**Figure 1:**
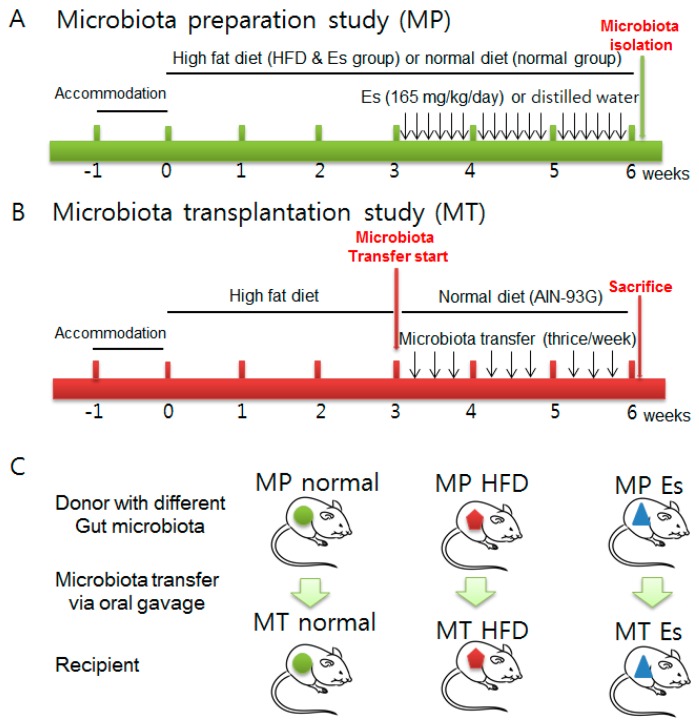
Experimental design microbiota preparation study (**A**) and microbiota transplantation study (**B**). The gut microbiota from microbiota preparation study were transplanted into its corresponding groups (**C**) as described in the Materials and Methods section. Abbreviations: MP, Microbiota preparation study; MT, Microbiota transplantation study; HFD, High-fat-diet; Es, *Ephedra sinica*.

The first step was the gut microbiota preparation study (MP) (Figure 1A): 24 male 6-week old Sprague-Dawley (SD) rats were purchased from Orient Bio (Seongnam, Korea). HFD and normal diet (AIN-93G, Table 1) were obtained from Feedlab (Guri, Korea). The animals were acclimatized for one week at a controlled temperature (20 ± 2 °C), relative humidity (40–60%) and a 12 h light-dark cycle with a commercial normal chow diet (Soyagreentec, Hwaseong, Korea) before the experiment. Rats were assigned randomly to three groups of eight each, as follows: MP normal, MP HFD, and MP Es group. Rats in the MP normal group were given the normal diet (AIN-93G, Table 1) ad libitum continuously for six weeks. Rats in the MP HFD and MP Es group were given commercial HFD with the same schedule as the MP normal group. Es (165 mg/5 mL/kg dissolved in distilled water) was administered orally by gavage once a day from the fourth week to sixth week in the Es group. DW was given at the same time and volume instead of Es in the normal and HFD groups.

All animals were weighed, fecal samples were collected before and after the experiments, and stored at −70 °C. After six weeks, blood samples were collected from the abdominal aorta under anesthesia. Animals were sacrificed, the abdominal and epididymal fat pads and liver tissue were collected, and stored at −70 °C. The fresh cecal contents were separately collected and kept at −80 °C for transplanting. Before transplanting, all cecal components in each MP group were separately pooled and dissolved with ten times the volume of phosphate buffered saline (PBS).

The second step was the gut microbiota transplantation study (MT, Figure 1B): the additional nine male SD rats (6 weeks, 180–220 g, Orient Bio, Seongnam-si, Korea) were divided into three groups: MT normal, MT HFD, and MT Es group. First, feces were collected both before and after MT in all groups. HFD is a strong inducer of obesity and can markedly alter the gut bacterial ecosystem at both the structural and functional level. To evaluate anti-obesity, the HFD supply was stopped and changed to a normal diet before gut microbiota transplantation. Afterwards, MT HFD group animals (*n* = 3) received microbiota collected from the HFD group, MT Es group animals (*n* = 3) received microbiota obtained from Es treated group animals, and normal MT group animals (*n* = 3) received microbiota obtained from normal group animals. Prepared MP derived gut microbiota were administered by oral gavage (10 mL/kg) three times per week for three weeks separately from the corresponding groups (Figure 1C). Eventually, the animals were weighed and sacrificed under anesthesia. Blood was collected from abdominal aorta and rapidly transferred into a BD Vacutainer (Franklin Lakes, NJ, USA). Subsequently, liver and fats were excised, weighed, rapidly dipped in physiological saline and stored at −80 °C for future use. After 2 h clotting, serum were separated by 3000× *g* centrifuge for 15 min and stored at −20 °C for future use. In both MP and MT, the volume of animal food intake was recorded by group. The Food Efficiency Ratio (FER) was computed by dividing the average body weight gain by the average food intake for each group.

### 2.4. Biochemical Analyses

Serum was separated using Vacutainer tubes (BD, Plymouth, UK) by 3000× *g* centrifuge for 15 min after blood clotting. The serum levels of aspartate transaminase (AST), alanine transaminase (ALT), fasting glucose, triglyceride (TG), total cholesterol (TC), and high density lipoprotein (HDL) were determined using an Auto Chemistry Analyzer (Hitachi, Tokyo, Japan).

### 2.5. Enzyme Immunoassay for Serum Insulin

Rat fasting serum insulin was measured using a Rat Insulin ELISA Kit (Mercodia, Uppsala, Sweden) according to the manufacturer’s directions. Briefly, 10 μL of samples or standards combined with 100 μL of enzyme conjugate solution were added to an antibody coated plate, followed by incubation for two hours at room temperature. After washing six times with 700 μL wash buffer solution per well, 200 μL substrate TMB was added, followed by incubation for 15 min at room temperature. Finally, 50 μL stop solution was added and the plate was read immediately on a spectrophotometer at 450 nm. The concentration of fasting serum insulin was calculated by standard curve. The homeostatic model assessment for insulin resistance (HOMA-IR) is commonly used as an index of insulin resistance in clinic and epidemiological study. In the current study, HOMA-IR scores were calculated from fasting glucose and insulin levels according to the previous method [25].

### 2.6. Fecal Acetic Acid Quantification by HPLC

Frozen fecal samples were dissolved in HPLC-grade water (Sigma) at a proportion of 1/4 (*w*/*v*). After sonication for 10 min and 13,000× *g* centrifugation for 3 min, 0.2 μm millipore filtered supernatants were stored at −70 °C. Prepared samples were analyzed using the YL9100 HPLC system (Young-Lin, Anyang, Korea) equipped with an Aminex-87H column (150 mm, 4.6 mm, Bio-Rad, Hercules, CA, USA) and YL 9120 UV/Vis detector (Young-Lin). Acetic acid (Sigma-Aldrich) was used as a standard. Samples were eluted with 0.008 N sulfuric acid at 0.6 mL/min for 30 min. All chromatograms were obtained using a wavelength of 215 nm and YL-Clarity was applied for calculating concentrations of fecal organic acids according to the standard curve.

### 2.7. Determination of Fecal Endotoxin

The Limulus Amebocyte Lysate (LAL) test is an official method for endotoxin detection regulated by the US Food and Drug Administration [26]. The levels of fecal endotoxin were determined using a LAL kit (ENDOSAFE, Charleston, SC, USA) according to the manufacturer’s protocol. PBS dissolved fecal supernatant samples were added into 5 EU/mL standard spiked or non-spiked wells in duplicate. Following addition of 100 μL LAL reagent, the kinetic absorbance of the mixture was measured at 405 nm and the reaction onset times of the samples were compared to the standard curve. The quality of data was confirmed in accordance with the spike recovery rates within 50% to 200%.

### 2.8. Real-Time Polymerase Chain Reaction (PCR)

Real-time PCR was used to determine the MCP-1, IL-1β and β-actin gene expression in liver tissue using following primers (MCP-1, forward 5′-CAG CCA GAT GCA GTT AAT GCC-3′, reverse 5′-AGC CGA CTC ATT GGG ATC AT-3′; IL-1β, forward 5′-CAC CTC TCA AGC AGA GCA CAG-3′, reverse 5′-GGG TTC CAT GGT GAA GTC AAC-3′; and β-actin, forward 5′-AGA GGG AAA TCG TGC GTG AC-3′, reverse 5′-CCA TAG TGA TGA CCT GTC CGT-3′). Total RNA was isolated using TRIsure reagent (Bioline, Taunton, MA, USA), followed by cDNA synthesis using the AccuPower RT premix kit (Bioneer, Daejeon, Korea). PCR was performed using a LightCycler FastStart DNA Master SYBR Green kit and a LightCycler instrument (Roche, Indianapolis, IN, USA). The standard conditions for the PCR amplification reactions were applied, as previously described [17]. The LightCycler software (Roche Applied Science) was used for analysis. Relative gene expression was represented by 2^−Δct^ calculations (ΔCt = Ct_target gene_ − Ct_β-actin_). The results are expressed as normalized fold values relative to the normal group.

### 2.9. Pyrosequencing and Bioinformatics Analysis

Fecal samples were collected and immediately stored at −70 °C. Genomic DNA was extracted using the QIAamp DNA feces mini kit (Qiagen, Hilden, Germany). Two or three samples from each group were selected for the pyrosequencing analysis. Pyrosequencing analyses were performed at Chun Lab (Seoul, Korea). The target region of the 16S rRNA genes (V1–V3 of a variable region was amplified using a C1000 Touch thermal cycler (Bio-Rad, Hercules, CA, USA) and purified using a QIAquick PCR purification kit (Qiagen). After quantification of purified products using the PicoGreends DNA Assay kit (Invitrogen, Carlsbad, CA, USA), equimolar concentrations of each amplicon were sequenced on a Roche/454 GS Junior system according to the manufacturer’s instructions. For analysis of pyrosequencing data, raw sequences for each sample were sorted according to their unique barcodes. Low quality reads (average quality scores <25 or read length <300 bps) were filtered and primer sequences were trimmed using pair-wise alignment and HMMER 3.0 package (HHMI Janelia Farm Research Campus, Ashburn, VA, USA). Trimmed sequences were corrected for sequencing errors by clustering, and representative sequences in each cluster were assigned their taxonomic position. Taxonomic identification was performed using the EzTaxon-e database according to the highest pair wise similarity among the top five BLASTN (Basic Local Alignment Search Tool Nucleotide) results. Possible chimera sequences were removed using the UCHIME. Operational taxonomic units picking, taxonomic assignment and phylogentic reconstruction were analyzed using QIIME (Version 1.9.1, University of Colorado, Boulder, CO, USA) software package and visualization in R Programming (Version 3.2.2, University of Auckland, Auckland, New Zealand). The functional and taxonomic difference of metagenomics data were predicted by PICRUSt-1.0.0 (Phylogenetic Investigation of Communities by Reconstruction of Unobserved States) [27] and HUMAnN2 (The HMP Unified Metabolic Analysis Network 2, http://huttenhower.sph.harvard.edu/humann2) [28]. The significant predicted results were statistically analyzed and represented graphically by STAMP v2.1.3 software (Dalhousie University, Halifax, Canada, http://kiwi.cs.dal.ca/Software/STAMP).

### 2.10. Statistical Analysis

All values were analyzed using one-way ANOVA followed by LSD (least significant difference) post-hoc test using the Statistical Package for Social Science (release 2008, SPSS Statistics for Windows, Version 17.0. SPSS Inc., Chicago, IL, USA) software. The results were expressed as the mean ± standard deviation (SD). Null hypotheses of no difference were rejected if *p*-values were less than 0.05.

## 3. Results

### 3.1. Es Extraction Yield and Ephedrine Proportion

**Figure 2:**
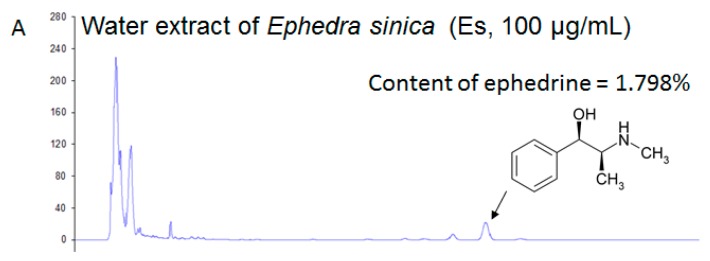

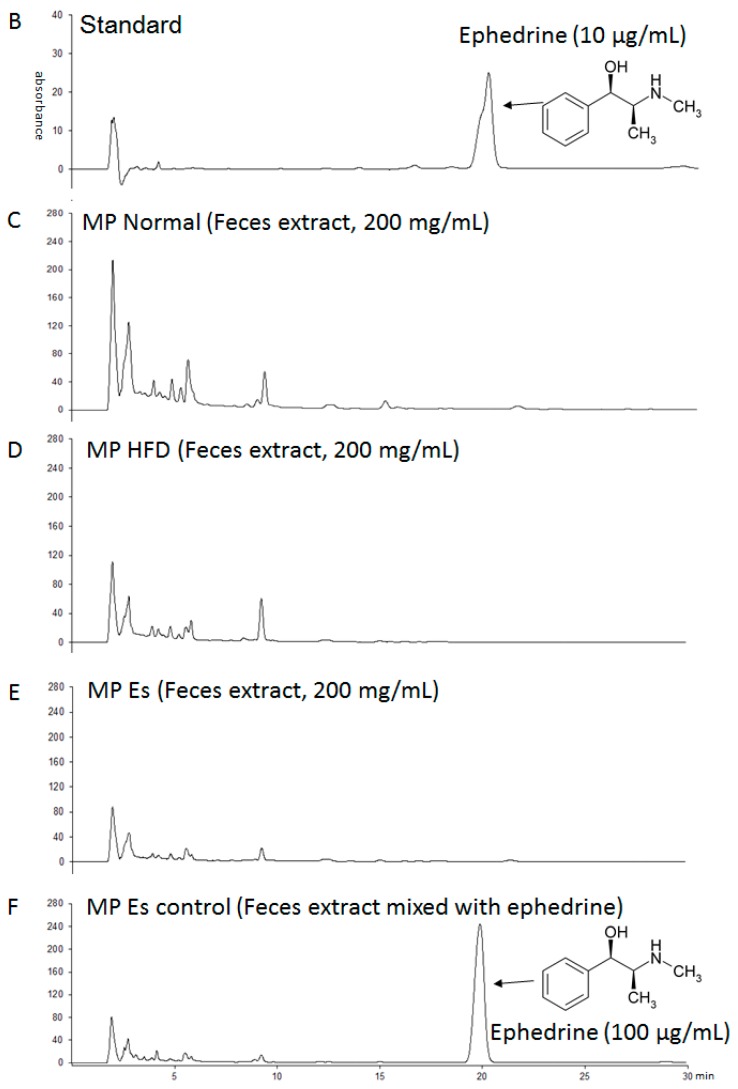
High-performance liquid chromatography (HPLC) fingerprinting of the water extract of *Ephedra sinica* (Es) and standards were filtered and subjected to HPLC analysis. (**A**) Water extract of Es (100 μg/mL), (**B**) standard: ephedrine (10 μg/mL), (**C**) MP normal fecal extract (200 mg/mL), (**D**) MP HFD fecal extract (200 mg/mL), (**E**) MP Es fecal extract (200 mg/mL), (**F**) MP Es control (MP Es fecal extract mixed with 100 μg/mL of ephedrine). The chromatograms were produced using a wavelength of 215 nm.

The final extraction of Es gave a yield of 14.83% (*w*/*w*). By calculating with a standard curve (R^2^ = 1, data not shown), the proportion of ephedrine in Es extract was approximately equal to 1.798% (*w*/*w*) (Figure 2A,B). Therefore, 29.7 mg/kg of ephedrine was administered in the MP study, while ephedrine was not detected in MP Es feces according to the HPLC results (Figure 2C–F).

### 3.2. Both Es and Es Altered Gut Microbiota Changed Body Weight (Gain), Fats and Organs Weight and Food Intake

Es treatment significantly reduced body weight (gain), and food intake compared to the MP HFD group. EsM showed the same pattern in accordance with Es extract. Both Es treatment and EsM significantly decreased the food intake compared to the corresponding HFD group. HFD caused a marked increase in fat weight, including abdominal fat, epididymal fat, and perirenal fat, compared to the MP normal group. Es treatment resulted in significantly decreased body weight, and body weight gain compared to the MP HFD group. EsM showed the same pattern in accordance with Es extract. Both Es extract and EsM influenced appetite compared to the corresponding HFD group (Table 2). Consequently, both HFD and HFD alteration of gut microbiota resulted in significantly increased FER compared to the corresponding normal group; however, Es and EsM did not change the HFD elevated FER.

### 3.3. EsM Ameliorated Hyperglycemia and Hyperinsulinemia, but Did Not Alter Serum Aminotransferases and Lipid Parameters

Except that HFD obviously elevated the serum ALT compared to the MP normal group, both Es and EsM did not notably change the level of serum AST and ALT. Similarly, HFD feed for six weeks significantly increased serum TG level compared to the normal diet, while Es treatment resulted in noticeably reduced serum TG level compared to the MP HFD group. However, in microbiota transplantation groups, the serum TG and TC level was not noticeably altered. HFD induced a prominent increase in FBG, serum insulin, and HOMA-IR compared to normal diet. Both Es and EsM resulted in markedly reduced FBG, serum insulin, and HOMA-IR (Table 3).

### 3.4. Both Es and EsM Ameliorated Enterogenous Endotoxin

**Figure 3:**
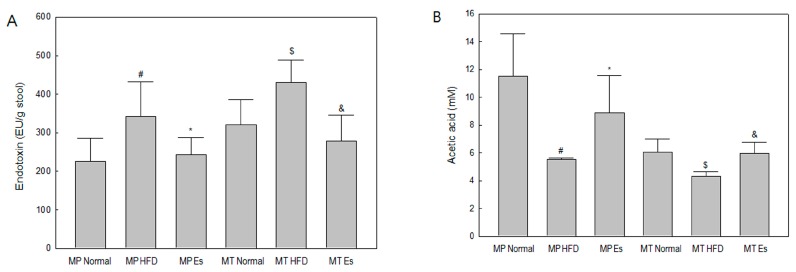
Quantitative determination of endotoxin and Acetic acid level in rat feces at the end of experimental schedule, the stool was collected from the animals. (**A**) The fecal endotoxin level was determined using a Limulus Amebocyte Lysate (LAL) kit. Data are expressed as mean ±SD (standard deviation). ^#^ *p* < 0.05 compared to the MP normal group; * *p* < 0.05 compared to the MP HFD group (*n* = 8); ^$^ *p* < 0.05 compared to the MT normal group; ^&^ *p* < 0.05 compared to the MT HFD group (*n* = 3); (**B**) The fecal acetic acid level was determined using the YL9100 HPLC system and an Aminex-87H column. HPLC chromatograms were obtained at a wavelength of 215 nm. Level of fecal acetic acids were calculated by YL-Clarity according to the standard curve.

HFD and HFD alteration of gut microbiota identically induced a significant increase in fecal endotoxin level compared to the corresponding normal group, while Es and EsM also identically resulted in evident diminution of the fecal endotoxin level compared to the corresponding HFD group (Figure 3A).

### 3.5. Both Es and EsM Elevated the Fecal Acetic Acid Level

HFD and HFD alteration of gut microbiota similarly reduced the acetic acid level in feces compared to the corresponding normal group. Both Es and EsM also led to marked restoration of the fecal acetic acid level compared to the corresponding HFD group (Figure 3B).

### 3.6. Both Es and EsM Down-Regulated Gene Expression of Key Proinflammatory Cytokines

**Figure 4:**
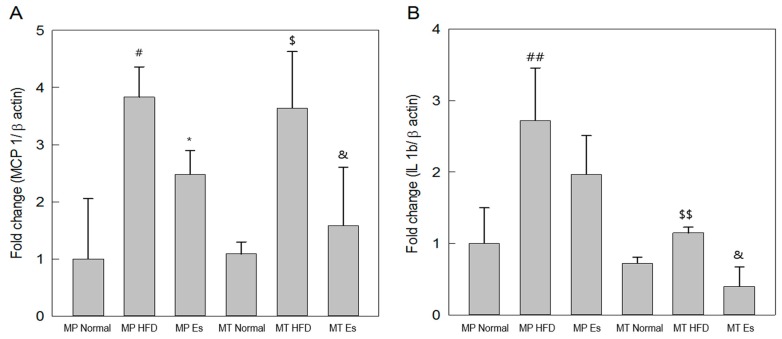
Real-time polymerase chain reaction (PCR) analysis. The gene expression of the MCP-1 (**A**) and IL-1β (**B**) were analyzed by real-time PCR in liver tissue. The results are expressed as normalized fold values relative to the MP normal group. ^#^ *p* < 0.05, ^##^ *p* < 0.01 compared to the MP normal group; * *p* < 0.05 compared to the MP HFD group (*n* = 8); ^$^ *p* < 0.05, ^$$^ *p* < 0.01 compared to the MT normal group; ^&^ *p* < 0.05 compared to the MT HFD group (*n* = 3).

HFD and HFD alteration of gut microbiota similarly resulted in significant up-regulation of the gene expression of MCP-1 and IL-1β compared to the corresponding normal group. Both Es and EsM resulted in down-regulation of *MCP-1* gene expression compared to the corresponding HFD group. Only EsM, not Es itself, significantly down-regulated gene expression of IL-1β compared to the corresponding HFD group (Figure 4A,B).

### 3.7. Gut Microbiota from Donor Evidently Influenced the Host Gut Microbial Community

**Figure 5:**
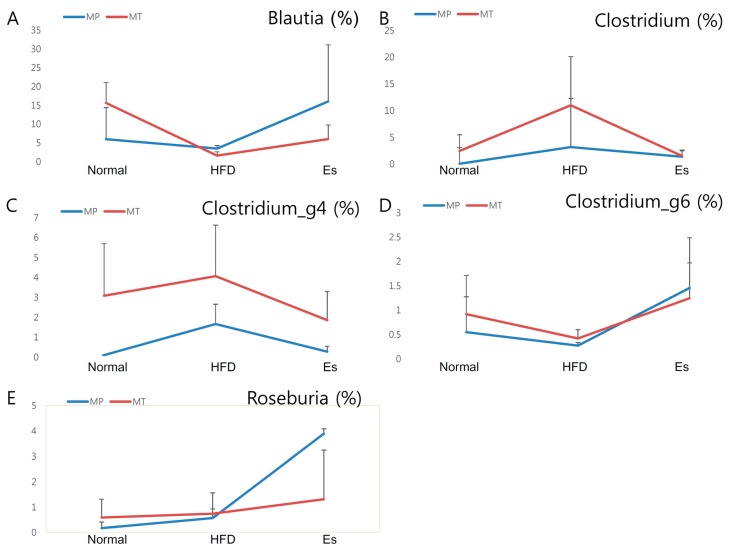

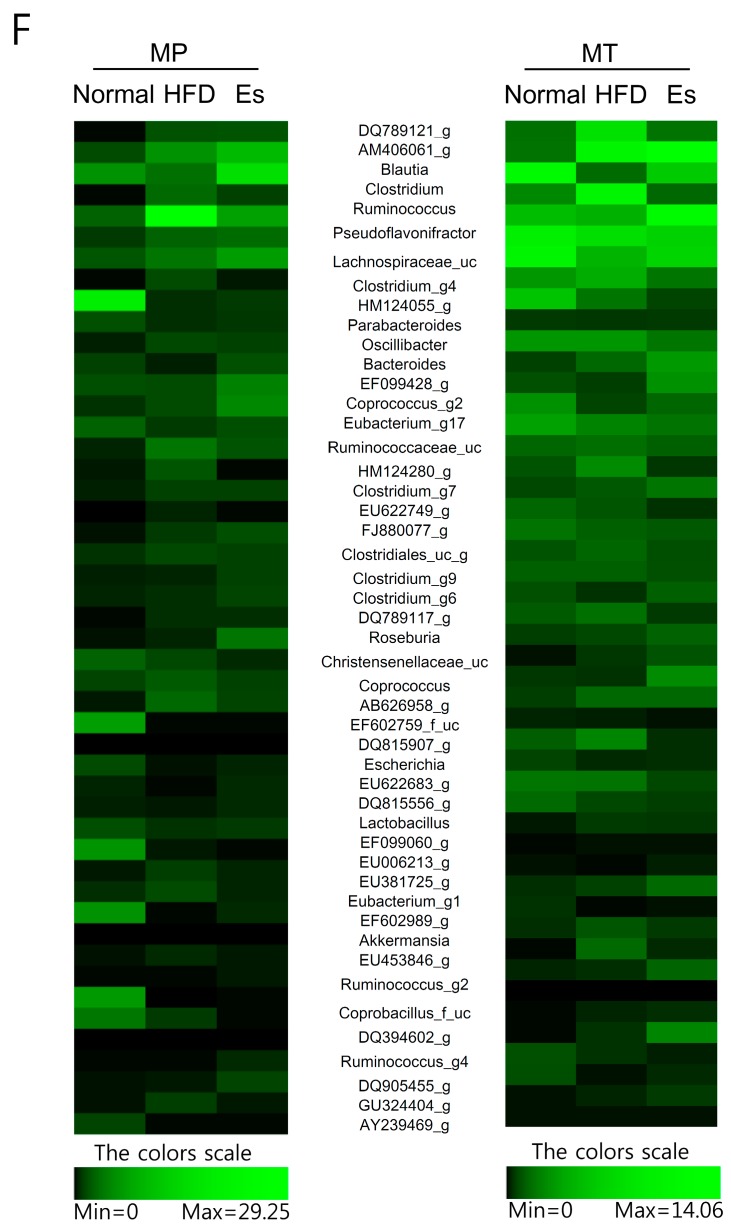
Comparison of genus level of gut microbiota At least more than 0.5% of genus of gut microbiota in any group were selected according to the pyrosequencing data. The variation tendency of several representative genera, like Blautia (**A**); Clostridium (**B**); Clostridium_g4 (**C**); Clostridium_g6 (**D**) and Roseburia (**E**), showed a conformity pattern between MP and MT experiment; (**F**) At least more than 1% of genus in any group were selected and compared using heatmap. The visualized heatmap were figured by PermutMatrix (Version 1.9.3 EN). As the colors scale shown, green color indicates a higher proportion, while black color shows a lower proportion.

More than 0.5% of family and genus in donor and recipient were selected for gut microbiota transplantation comparability checking (Figure 5). At family level, 61.54%, 84.62%, and 71.43% of donors were in accordance with their corresponding receptor. Similarly, 45.71%, 70.27%, and 75.68% of donors were in accordance with their corresponding recipient at genus level (Figure S1).

### 3.8. Predicted Alteration of Gut Microbiome Function

**Figure 6:**
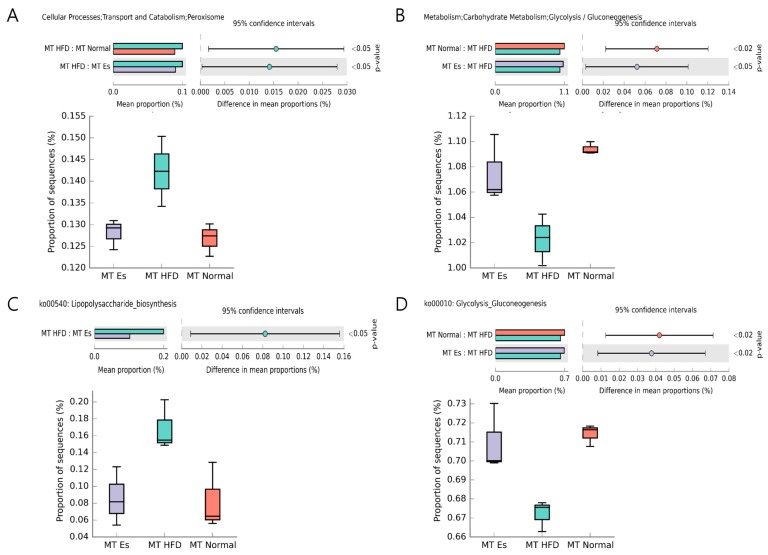
Predicted difference of gut microbiome function and KEGG pathway. The significant difference (*p* < 0.05, confidence intervals equal 95%) of PICRUSt (**A**,**B**) and HUMAnN2 (**C**,**D**) derived predictive finding was figured by STAMP (Version 2.1.3).

PICRUSt and HUMAnN2 pipelines were applied to predictive functional and taxonomic profiling of gut microbial communities using 16s rRNA gene sequences. HFD altered gut microbiota markedly increased the peroxisome and decreased the glycolysis/gluconeogenesis as compared to MT normal group. However, EsM remarkably suppressed these changes compared to MT HFD group (Figure 6A,B). The functional pathway predicted results showed that EsM significantly changes the lipopolysaccharide biosysnthesis pathway (ko00540) and glycolysis/gluconeogenesis pathway (ko00010) compared to MT HFD group (Figure 6C,D).

## 4. Discussion

In both developed and developing countries, obesity has been rapidly increasing for the past 30 years [29]. More than 2.1 billion people (approximately one third the world’s population) are overweight and obese worldwide until 2013 [29]. Unhealthful dietary habits, like high fat diet, directly change the normal distribution of gut microbiota following the reduction of intestinal microbiota diversity [13,30]. Dysfunction of gut microbiota evidently promotes development of chronic metabolic disorders [31] via regulation of energy homeostasis and fat storage [32]. Therefore, modulating intestinal microbiota was deemed a feasible strategy for treatment of obesity and related diseases. Our previous studies verified that medicinal herbs effectively ameliorate obesity and metabolic endotoxemia associated with regulation of gut microbiota distribution and gut permeability in an animal metabolic disorder model [17,33].

HFD alters gut microbiota, and can directly cause obesity independent of dietary type in a gnotobiotic mouse model [34]. Es exerted a beneficial effect on obesity and glucose intolerance in a HFD mice model [7]. Our previous study demonstrated that although the individual sensitivity to *ephedra* differs, alteration of gut microbiota by *Ephedra* intake was associated with loss of BW and BMI in human subjects [24]. In the MP, three types of gut microbiota (from normal, HFD, or HFD together with Es treatment, respectively) were transplanted by oral gavage to 3-week HFD fed rats. It is noteworthy that ephedrine was not detected even in Es treatment fecal samples and there was no obvious difference among the three types of MP feces extract according to the HPLC profiling. Thereby it is sufficient to exclude the transplantation effect caused by ephedrine. Though the antibiotics can exceedingly disrupt intestinal microbial ecology, it seems more facile to transplant gut microbiota from donor, however none scientific evidence revealed antibiotics pretreatment before fecal transplantation is more beneficial than non-treatment in duplication of donor phenotypes and bacterial phylotypes, therefore in the current study we did not use any antibiotics before transplantation to eliminate the preexisting gut bacteria. The conventional recipient is more similar to a clinical obesity model than a gnotobiotic model with physiological abnormalities. As expected, Es administration obviously reduced the body weight (gain), total visceral weight and food intake compared to the HFD control group. Commonly Es is considered an appetite suppressant for weight loss due to its *Ephedra* alkaloid compounds. Notably our data showed that EsM transplantation reduces body weight, total visceral weight and intake of food, without interfering with FER. The above results indicate that appetite suppression by EsM transplantation is a potential anti-obesity mechanism.

A short-chain fatty acid acetate that can directly reduce appetite via regulation of central homeostasis was reported [35]. Many gut bacterial species, including *Lactobacillus* spp., can promote acetate production in HFD-fed mice [36], and acetate suppresses body fat accumulation in rats [37]. High levels of fecal acetic acid were observed in both the Es treatment group and EsM transplantation group. This indicates that EsM maintained the body weight and visceral fat weight loss effect through augmentation of the short chain fatty acid acetate, bringing about the anorexigenic property.

In addition, to investigate the change of visceral fat in detail, the weights of mesenteric, epididymal, and perirenal fat were compared separately. Interestingly, Es administration resulted in noticeably decreased mesenteric and epididymal fat weight compared to HFD control. However, EsM more obviously decreased epididymal fat and perirenal fat weight. Es and EsM showed a partial difference in characteristics of visceral fat distribution. Es administration did not reduce the weight of fat liver increased by HFD. Both HFD altered gut microbiota (HFM) and EsM did not influence liver weight. Serum aminotransferases also did not change in any microbiota transplantation groups. Although the microbiota alteration influencing lipid and glucose metabolism has been demonstrated in an ob/ob mice model [38], in our experiment alteration of gut microbiota transplantation resulted in amelioration of fasting serum glucose but not lipid profile, such as TG, TC, and HDL. This might be due to a short period (three weeks) of microbiota transplantation and individual differences of SD rats. Antibiotic alteration of gut microbiota improves insulin resistance in diet-induced obesity [39]. In the current experiments, both Es and EsM transplantation resulted in improvement of insulin resistance, as evidenced by inhibition of serum insulin concentration and HOMA-IR. Besides, EsM significantly enhanced glycolysis and gluconeogenesis through influence of metabolic pathway (ko00010) (Figure 6B,D). It is probable evidence for improving hyperglycemia and hyperinsulinemia associated with gut microbiota change.

**Figure 7:**
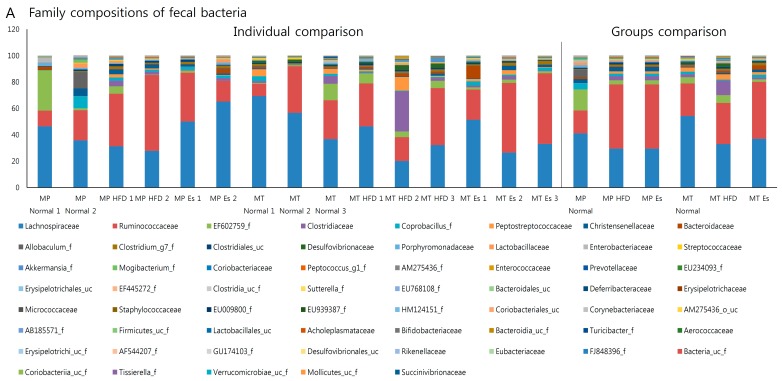

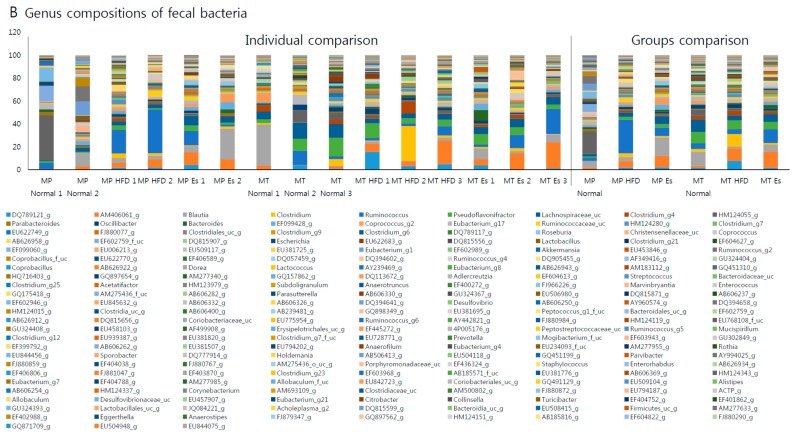
Gut microbiota taxonomic profiles. The relative contributions (%) of fecal bacterial family (**A**) and genus (**B**) were compared both individually and averagely according to the pyrosequencing data.

HFD significantly affects the gut microbiota ecosystem at the functional level [30]. HFD feeding decreased the weight of cecum contents via alteration of intestinal bacteria metabolism while antibiotic treatment significantly increased it [13]. In the current experiment, both Es and EsM resulted in markedly augmented HFD lowered weight of cecum contents. The effect of the cecal weight by Es or ESM might involve a close association with the impact of microbiota diversity and composition. The PCoA analysis did not show an obviously different pattern of gut microbiota composition in each group (Figure S2). However, family and genus level of gut microbiota were similar between donor and recipient (Figure 5, Figure 7 and Figure S1).

Low-grade inflammation triggered by gut microbiota-derived endotoxin, leading to initiation of obesity and insulin resistance was confirmed [12]. HFD induced significant elevation of the fecal endotoxin although Es notably prevented the increase. It is noteworthy that gut microbiota transplantation groups showed a similar pattern of fecal endotoxin compared to each donor. It might be that the pattern of gut microbiota is closely associated with the intestinal endotoxin environment. In accordance with fecal endotoxin pattern, KEGG pathway predictions from HUMAnN2 based on 16s rRNA bacteria gene sequences indicated that EsM significantly negatively impact on LPS biosynthesis pathway (ko00540). Enteral endotoxin induced inflammation was evidenced by high expression of IL-1 beta and MCP-1 cytokines. As demonstrated, IL-1β is a quintessential pro-inflammatory cytokine associated with obesity and metabolic disorders [40], and MCP-1 accelerates macrophage infiltration in inflammation accompanying obesity [7]. Up-regulation of gene expression of MCP-1 was found in adipose tissue of obese subjects compared to lean control [41]. Both Es and EsM resulted in significantly down-regulated gene expression of MCP-1 compared to HFD control. This indicates that the endotoxin-induced inflammation was inhibited by alteration of gut microbiota.

**Figure 8:**
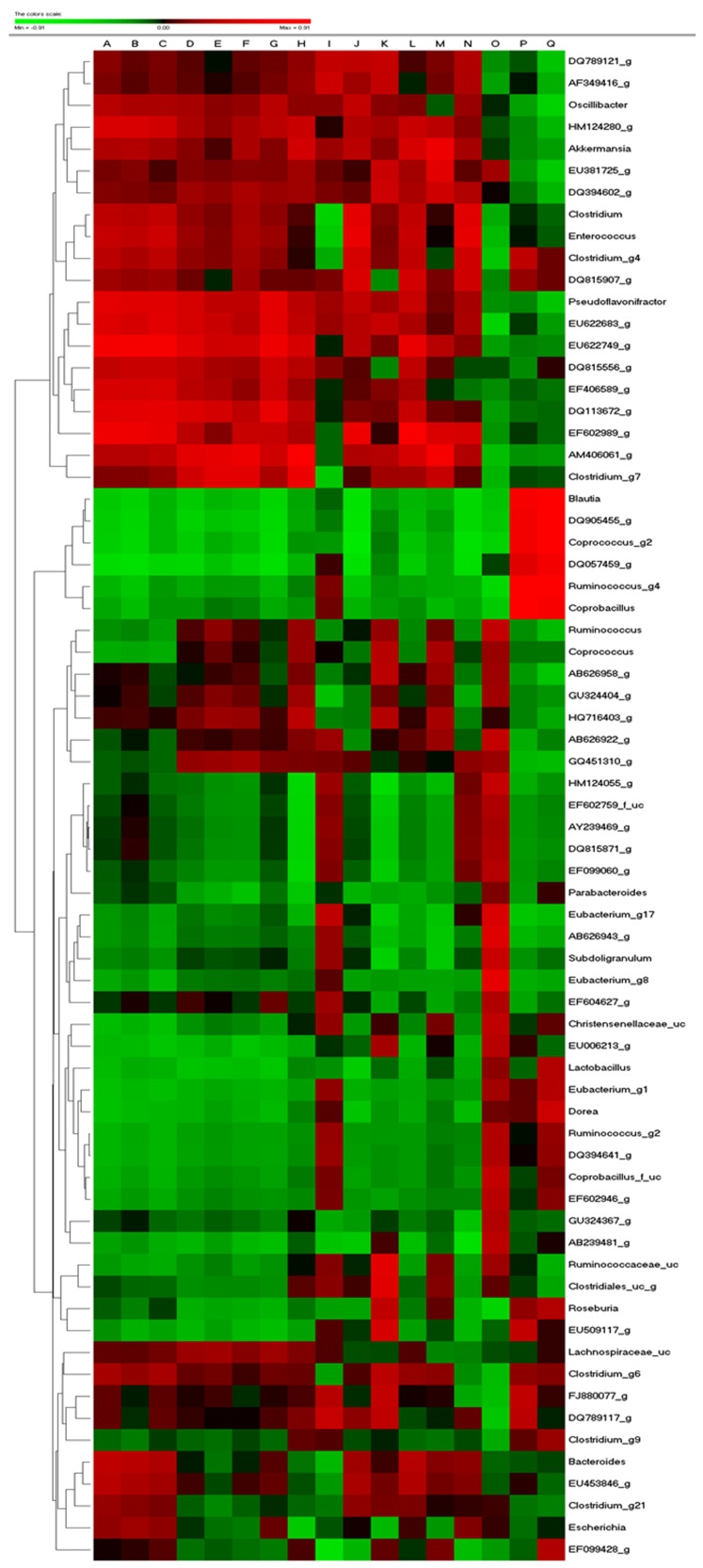
Pearson correlation analysis. All of the data were collected and analyzed using Pearson's correlation score. The scores of Pearson’s correlation were figured by PermutMatrix software (Version 1.9.3 EN) using heatmap plots. As the colors scale shown, red color indicates a positive correlation, while green color shows a negative correlation. A: Body weight; B: Body weight gain; C: FER (Food efficiency ratio); D: Total fat; E: AFAT (Abdominal fat); F: EFAT (Epididymal fat); G: PFAT (Perirenal fat); H: Liver; I: AST (aspartate transaminase); J: ALT (alanine transaminase); K: TC (total cholesterol); L: TG (triglyceride); M: FBG (fasting blood-glucose); N: Insulin; O: Food intake; P: HDL (high density lipoprotein); Q: Cecum contents.

In detail, gut microbiota pyrosequencing data showed similar trends at genus level. Blautia, Roseburia, and Clostridium_g6 were selectively enriched by Es and EsM. In particular, most species of Blautia are regarded as short chain fatty acid producers [42]. Hence the enrichment of Blautia is a possible reason for the high level of fecal acetic acid leading to appetite-reduction and eventual weight loss. Blautia is negatively associated with body weight, fat weight, and FBG, different from other genus of gut microbiota (Figure 8). Roseburia was also found to show negative correlation with body weight, consistent with a previous study [43]. In addition, Akkermansia ameliorates HFD induced obesity, metabolic endotoxemia, and glucose homeostasis via change of microbial metabolites, such as SCFA and interference of Foxp3 regulatory T cells [44,45]. Our experiment showed that EsM transplantation maintained the restoration of Akkermansia abundance loss. This might be another mechanism to explain why the anti-obesity effect can be maintained only by alteration of gut microbiota transplantation.

Besides the abovementioned bacteria, body and fat mass were negatively associated with Coprococcus, Ruminococcus, Eubacterium, dorea, and positively associated with Oscillibacter, Chlostridium, Bacteroides, Escherichia. Because of the complex interaction between the variety of gut microbiota and host metabolism [46], multivariate regression analysis is a feasible approach to search for the potential indicators mechanistically connected between the cause and the disease [14]. Therefore, in future it will be worthwhile to confirm if and how these associated bacteria regulate the development of obesity and metabolic disorders via germ free and/or transgenic model. In the MT, normal gut microbiota (NM) was used as a positive control for monitoring transplantation response. Interestingly, both EsM and NM transplant exert the similar amelioration efficacy on HFD induced obesity and metabolic disorders. NM transplant improved most of the metabolic parameters to a greater extent than EsM transplant while EsM transplant was more effective in epididymal fat than NM transplant. As limitation of the study, because the group of normal diet with Es treatment is absent, we are not able to confirm whether normal diet with Es exert synergistic effect via gut microbiota regulation.

## 5. Conclusions

EsM transplantation preserves the amelioration efficacy on HFD induced obesity and metabolic disorders by the association of modulating the distribution of intestinal microbiota, enterogenous endotoxin and enteral acetic acid.

## Figures and Tables

**Table 1:** Animal diets formulation and ingredients.

Formulation	Normal Diet (AIN-93G)		High Fat Diet	
	g%	kcal%	g%	kcal%
Protein	20	20	27	20
Carbohydrate	64	64	25	20
Fat	7	16	36	60
kcal/kg	4000		5333	
**Ingredient**	**g**	**kcal**	**g**	**kcal**
Casein (from milk)	200	800	200	800
Corn starch	397,486	1590	47,536	190
Sucrose	100	400	0	0
Dextrose	132	528	132	528
Cellulose	50	0	50	0
Soybean oil	70	630	25	225
Lard	0	0	245	2205
Mineral mixture	35	0	35	0
Vitamin mixture	10	40	10	40
TBHQ	0.014	0	0.014	0
l-Cystine	3	12	3	12
Choline bitartrate	2.5	0	2.5	0
Total	1000	4000	750.1	4000

Abbreviations: TBHQ, tertiary butylhydroquinone.

**Table 2:** Comparison of body, fat and organ weights, food intake and food-efficiency ratio.

	Microbiota Preparation Experiment (MP)			Microbiota Transfer Experiment (MT)		
Groups	MP Normal	MP HFD	MP Es	MT Normal	MT HFD	MT Es
Initial body weight (g)	244.0 ± 9.6	248.0 ± 7.7	244.9 ± 10.2	271.1 ± 3.3	268.2 ± 4.9	272.9 ± 6.0
Final body weight (g)	444.9 ± 20.3	553.6 ± 61.7 ^#^	487.9 ± 56.8 *	524.6 ± 21.5	587.3 ± 5.6 ^&^	532.8 ± 14.1 ^$^
Body weight gain (g)	200.9 ± 23.8	305.6 ± 59.9 ^#^	249.1 ± 42.9 *	253.5 ± 18.2	319.1 ± 10.3 ^&^	260.0 ± 12.0 ^$^
Food intake (g/day)	23.8 ± 2.5	22.0 ± 2.8	18.1 ± 3.7 *	25.3 ± 0.8	22.1 ± 0.8 ^&^	18.7 ± 1.2 ^$^
Food efficiency ratio	0.21 ± 0.02	0.35 ± 0.08 ^#^	0.36 ± 0.11	0.24 ± 0.01	0.34 ± 0.00 ^&^	0.33 ± 0.01
Total fat (g)	46.0 ± 4.9	82.3 ± 19.2 ^#^	63.1 ± 6.7 *	52.5 ± 9.8	83.1 ± 5.9 ^&^	58.5 ± 2.8 ^$^
Relative of total fat (%)	7.89 ± 0.66	11.75 ± 2.90 ^#^	10.06 ± 0.73	10.0 ± 1.5	14.2 ± 1.1 ^&^	10.9 ± 0.4 ^$^
Abdominal fat (g)	10.8 ± 1.4	17.9 ± 5.1 ^#^	13.1 ± 2.3 *	11.3 ± 4.1	17.1 ± 1.4	16.3 ± 2.0
Epididymal fat (g)	9.8 ± 0.9	17.8 ± 3.9 ^#^	13.6 ± 2.1 *	17.3 ± 2.4	28.0 ± 1.8 ^&^	15.0 ± 1.2 ^$^
Perirenal fat (g)	14.5 ± 2.4	28.6 ± 5.9 ^#^	22.5 ± 3.4	23.9 ± 4.0	38.0 ± 4.1 ^&^	27.2 ± 4.5 ^$^
Liver weight (g)	12.9 ± 1.5	16.4 ± 2.1 ^#^	14.1 ± 2.3	15.0 ± 0.8	15.2 ± 0.7	15.5 ± 1.6
Relative of Liver weight (%)	2.91 ± 0.34	3.00 ± 0.56	2.88 ± 0.26	2.9 ± 0.1	2.6 ± 0.1 ^&^	2.9 ± 0.3
Cecum weight (g)	5.6 ± 0.8	4.6 ± 0.2 ^#^	5.0 ± 0.3 *	4.2 ± 0.1	4.6 ± 0.1 ^&^	5.3 ± 1.3
Relative of Cecum weight (%)	1.26 ± 0.2	0.84 ± 0.08 ^#^	1.04 ± 0.09 *	0.80 ± 0.04	0.79 ± 0.02	0.99 ± 0.27
Cecum contents (g)	1.90 ± 0.57	1.39 ± 0.19 ^#^	1.69 ± 0.21 *	1.7 ± 0.2	1.1 ± 0.1 ^&^	1.5 ± 0.2 ^$^

Data are expressed as mean ± SD and statistically evaluated using one way ANOVA followed by Least Significant Difference (LSD) pos-hoc test. ^#^ *p* < 0.05, compared to the MP normal group; * *p* < 0.05 compared to the MP HFD group (*n* = 8); ^&^ *p* < 0.05, compared to the normal MT group; ^$^ *p* < 0.05 compared to the MT HFD group (*n* = 3). Relative tissues weight (%) = (tissue weight/final body weight) × 100%. Food efficiency ratio = body weight gain (g/day)/food intake (g/day).

**Table 3:** Comparison of serum biochemical parameters.

	Microbiota Preparation Experiment (MP)			Microbiota Transfer Experiment (MT)		
Groups	MP Normal	MP HFD	MP Es	MT Normal	MT HFD	MT Es
AST (IU/L)	76.8 ± 10.8	73.9 ± 13.7	80.4 ± 14.1	79 ± 7.8	151.3 ± 41.2	113.0 ± 26.0
ALT (IU/L)	23.3 ± 4.2	35.4 ± 5.6 ^#^	35.1 ± 10.3	30.0 ± 6.2	32.3 ± 2.9	31.7 ± 2.5
TG (mg/dL)	28.8 ± 1.3	59.0 ± 12.1 ^#^	43.2 ± 6.1 *	43.0 ± 6.1	54.0 ± 11.4	39.7 ± 13.6
TC (mg/dL)	47.9 ± 7.8	52.9 ± 5.3	59.3 ± 11.9	65.7 ± 7.4	66.0 ± 4.6	59.3 ± 9.5
HDL (mg/dL)	28.6 ± 11.2	24.3 ± 2.0	33.1 ± 4.3 *	35.2 ± 4.1	29.7 ± 4.6	28.7 ± 5.3
FBG (mg/dL)	135.8 ± 27.5	186.4 ± 29.8 ^#^	145.1 ± 25.6 *	158.7 ± 18.5	268.7 ± 12.9 ^&^	190.0 ± 21.4 ^$^
Insulin (ng/mL)	0.28 ± 0.13	0.65 ± 0.14 ^#^	0.52 ± 0.07 *	0.24 ± 0.15	0.57 ± 0.11 ^&^	0.28 ± 0.08 ^$^
HOMA-IR (mg/dL)	0.09 ± 0.04	0.29 ± 0.05 ^#^	0.18 ± 0.04 *	0.11 ± 0.02	0.38 ± 0.09 ^&^	0.11 ± 0.06 ^$^

Abbreviations: AST, aspartate transaminase; ALT, alanine transaminase; TC, total cholesterol; HDL, high density lipoprotein; TG, triglyceride; FBG, fasting blood-glucose. Data were expressed as mean ± SD and statistically evaluated using one-way ANOVA followed by LSD post-hoc test. ^#^ *p* < 0.05 compared to the MP normal group; * *p* < 0.05 compared to the MP HFD group (*n* = 8); ^&^ *p* < 0.05 compared to the MT normal group; ^$^ *p* < 0.05 compared to the MT HFD control group (*n* = 3).

## Supplementary Materials

The following are available online at www.mdpi.com/1660-4601/14/6/555/s1, Figure S1: Comparison of gut microbiota, Figure S2: Principal coordinate analysis (PCoA) plots, Figure S3: Rarefaction curves, Table S1: Fecal endotoxin concentration and quality control.
