# Supplementary material for: Ephedra-Treated Donor-Derived Gut Microbiota Transplantation Ameliorates High Fat Diet-Induced Obesity in Rats
Source: Int J Environ Res Public Health. 2017 May 23;14(6):555. doi: 10.3390/ijerph14060555 (PMC5486241; doi:10.3390/ijerph14060555)

Ephedra -Treated Donor-Derived Gut Microbiota Transplantation Ameliorates High Fat Diet-Induced Obesity in Rats

Jing-Hua Wang, Bong-Soo Kim, Kyungsun Han and Hojun Kim

Figure S1. Comparison of gut microbiota

At least more than 0.5% of genus (A) and family (B) of gut microbiota in any group were selected according to the pyrosequencing data. The Venn diagrams were used to compare the similarity of gut microbiota between donor and recipient.

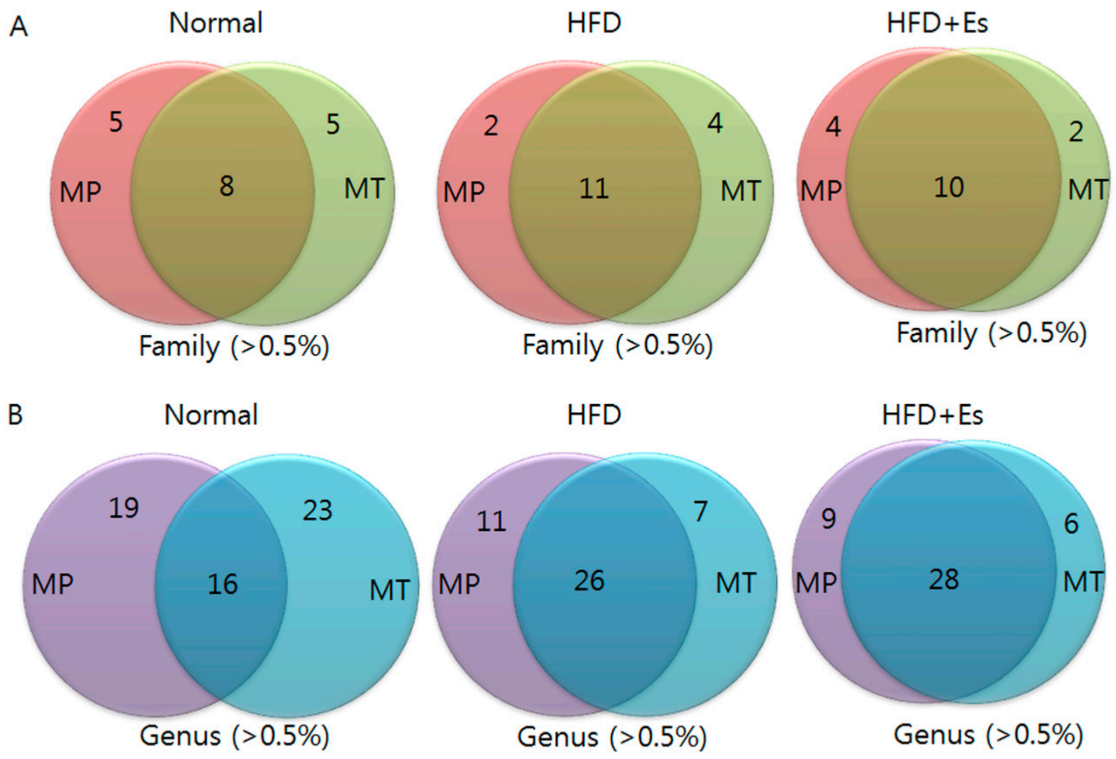

Figure S2. Principal coordinate analysis (PCoA) plots

PCoA plots are based on unweighted UniFranc distance according to fecal bacterial pyrosequencing data. Each point means a sample from MP fecal samples (A) and MT fecal samples (B). Green round represents sample from normal groups, red pentagon represents sample from HFD groups and blue hexagon represents sample from Es groups.

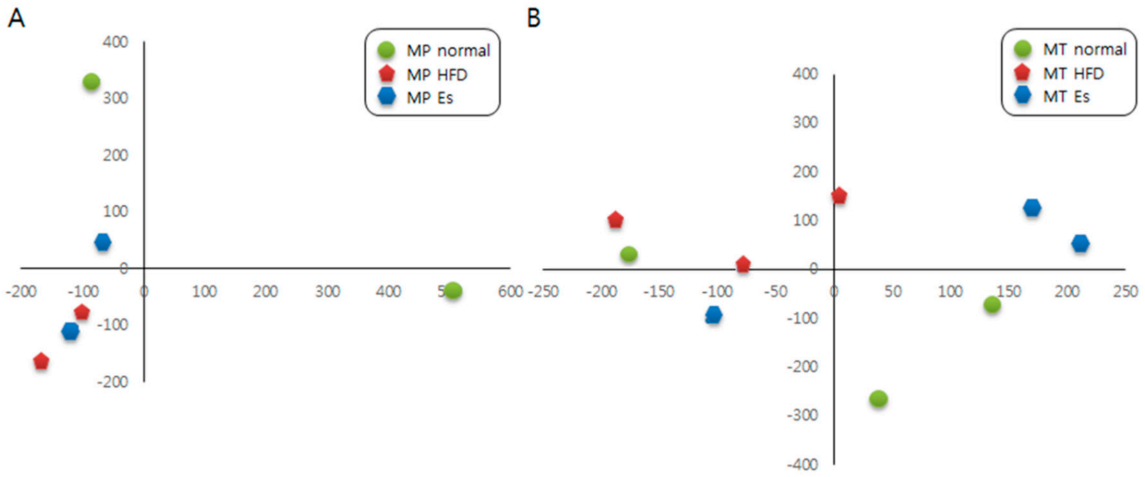

Figure S3. Rarefaction curves

The rarefaction curves represented for all samples comparison based on the pyrosequencing data. The rarefaction curves showed good sampling depth because of the near plateau phase achievement.

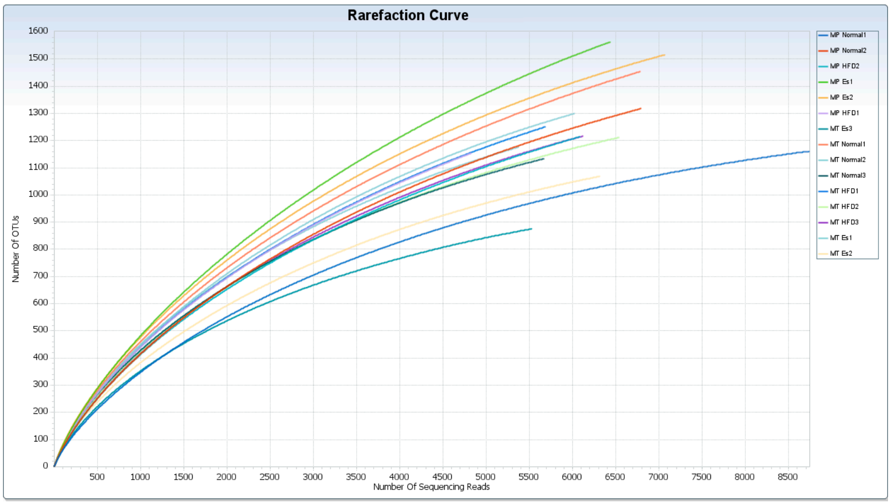

Supplement: Supplementary file 1 [file ijerph-14-00555-s001.zip › ijerph-187696-supplementary/ijerph-187696-supplementary.pdf]
